# RIOK1: A Novel Oncogenic Driver in Hepatocellular Carcinoma

**DOI:** 10.1002/cam4.70597

**Published:** 2025-01-25

**Authors:** Chunyan Ruan, Tianyu Shang, Sijia Zhang, Wenhong Ru, Yuefeng Yang, Yi Shen

**Affiliations:** ^1^ Centre for Medical Research Ningbo No.2 Hospital Ningbo China; ^2^ Guoke Ningbo Life Science and Health Industry Research Institute Ningbo China

**Keywords:** cell proliferation, hepatocellular carcinoma, multi‐omics analysis, potential effectors, RIOK1

## Abstract

**Background:**

Hepatocellular carcinoma (HCC) is one of the most common and highly lethal cancers worldwide. RIO kinase 1 (RIOK1), a protein kinase/ATPase that plays a key role in regulating translation and ribosome assembly, is associated with a variety of malignant tumors. However, the role of RIOK1 in HCC remains largely unknown.

**Methods:**

Changes in RIOK1 expression in HCC and patient prognosis were evaluated using HCC tissues and public databases. The functional role of RIOK1 in HCC was analyzed by RTCA assay, clonogenic assay, and flow cytometry in vitro, and by mouse tumor xenograft model in vivo. Potential mechanism studies were performed using multi‐omics analysis, public database screening, and qRT‐PCR assay.

**Results:**

In this study, we found that RIOK1 was elevated in HCC tissues and correlated with poor prognosis. Functional assays demonstrated that RIOK1 knockdown suppressed HCC cell proliferation, survival, and tumor growth in vivo, while RIOK1 overexpression enhanced these oncogenic phenotypes. Meanwhile, RIOK1 knockdown affected cell cycle progression and the expression of cyclin A2 and cyclin B1. Furthermore, integrated transcriptomic and proteomic analysis revealed that RIOK1 may promote HCC cell proliferation by affecting the cell cycle and DNA repair pathways. Moreover, we identified five potential effectors regulated by RIOK1: PMS1, SPDL1, RAD18, BARD1, and SMARCA5, which were highly expressed in HCC tissues and negatively correlated with the overall survival of HCC patients.

**Conclusion:**

Our findings suggest that RIOK1 is a novel oncogenic driver that may serve as a potential diagnostic and therapeutic target for HCC.

## Graphical abstract

This study reveals that the atypical protein kinase RIOK1, found to be elevated in hepatocellular carcinoma (HCC) tissues, may promote HCC cell proliferation by affecting the cell cycle and DNA repair pathways. Five potential effectors were identified, which were highly expressed in HCC tissues and negatively correlated with patient survival. Our findings suggest RIOK1 may serve as a novel diagnostic and therapeutic target for HCC.

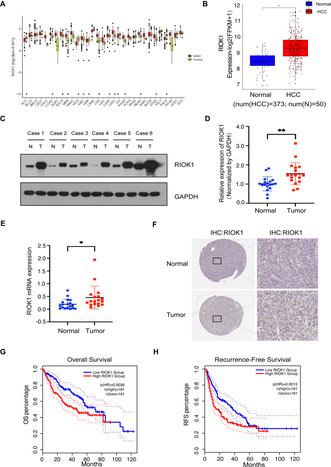

## 1 Introduction

Hepatocellular carcinoma (HCC), accounting for up to 90% of primary liver cancers worldwide, is a prevalent malignancy with high morbidity and mortality [1]. It is the fourth most common malignant tumor in China. Globally, the incidence of HCC continues to increase, while treatment options remain limited. Therefore, it is of great practical significance to elucidate the mechanisms underlying the occurrence and development of hepatocellular carcinoma and identify effective treatment strategies.

RIOK1 is an important member of the RIO kinase family, which also includes RIOK2 and RIOK3 [2, 3]. RIOK1 comprises kinase and ATPase activities. Unlike typical protein kinases, members of the RIOK family possess weak kinase activity and lack substrate binding sites and activation loop structures [4, 5]. Moreover, RIOK1 participates in regulating many important cell biological processes, including cell proliferation, cell survival, and gene transcription [6]. Previous studies have reported that RIOK1 is overexpressed in various solid tumors, which is associated with malignant phenotypes [7, 8, 9, 10]. For example, in non‐small cell lung cancer (NSCLC), high expression of RIOK1 can promote tumor cell proliferation, suppresse apoptosis, and facilitate invasion and metastasis [6, 7]. In HR‐negative breast cancer cells, overexpression of RIOK1 promotes cell growth and invasion by altering PI3K/AKT and MAPK/ERK signaling [10]. In addition, RIOK1 mediates p53 degradation by phosphorylating G3BP2 in colorectal cancer [11]. Although RIOK1 is associated with various solid tumors, its exact role and downstream signaling pathways in the pathogenesis of HCC remain unclear.

In this study, we report the role mediated by RIOK1 in hepatocellular carcinoma. We found that the high expression of RIOK1 in HCC tissues is positively correlated with HCC progression, and its overexpression is associated with poor prognosis. Using cellular models, we explored the effects of RIOK1 knockdown on HCC phenotypes, including cell proliferation, survival, and the cell cycle. Through correlation analysis of transcriptomic and proteomic data, we identified critical signaling pathways and potential effectors regulated by RIOK1. Thus, we hope to provide new insights for the early diagnosis and treatment of HCC.

## 2 Materials and Methods

### 2.1 Tissue Samples and Cell Lines

Eighteen HCC samples were obtained from 18 patients, along with matching non‐tumor liver tissues, following surgical resection at Ningbo No. 2 Hospital. This study was approved by the Human Research Ethics Committee of Ningbo No. 2 Hospital, and written informed consent was obtained from all the patients.

Human liver cell lines Huh7 and HepG2 were obtained from the Shanghai Cell Bank of the Chinese Academy of Sciences, China. HepG2 and Huh7 were cultured in DMEM (Hyclone, USA) supplemented with 10% FBS (Gibco, USA). The cells were maintained at 37°C in a humidified atmosphere containing 5% CO_2_. All cell lines were authenticated by the National Collection of Authenticated Cell Cultures (Shanghai, China) and confirmed to be mycoplasma negative.

### 2.2 Plasmids

The shRNA RIOK1 plasmid (shRIOK1#1 and shRIOK1#2) was constructed using pLKO.1‐mCherry‐Puro, while the RIOK1 overexpression plasmid was generated using pEZ‐Lv102‐Puro.

The shRNA RIOK1 sequence#1 was 5′‐GATCCAACACCTGTTCTACAGTTGCTCTG‐3′; the shRNA RIOK1 sequence#2 was 5′‐GAGACTTGAAGACAGTCAAAG‐3′; and the shRNA control sequence was 5′‐TTCTCCGAACGTGTCACGT‐3′.

### 2.3 Lentivirus Packaging and Infection

Lentiviral particles were generated in HEK293T cells by transient co‐transfection of the RIOK1 lentiviral expression plasmid along with the packaging plasmids pMD2G and pSPAX2 (MiaoLing, China) using Lipofectamine 3000 (Gibco, USA). Forty‐eight hours post‐transfection, the resultant lentiviral particles were used to transduce HepG2 or Huh7 cells in the presence of 8 μg/mL polybrene (Sigma, USA). Stable RIOK1 knockdown or overexpression cell lines were established through selection with a medium containing 2 μg/mL puromycin (Calbiochem, Germany).

### 2.4 RNA Isolation and Quantitative Real‐Time PCR

Total RNA extraction from the cells was performed with the TransZol Up Plus RNA Kit (Transgene, China). The purity and concentration of all extracted RNA samples were evaluated by measuring absorbance at 260/280 nm. Subsequently, first‐strand cDNA synthesis from the total RNA was undertaken using oligo dT primers and the Transcriptor First Strand cDNA Synthesis Kit (Roche, Switzerland). Quantitative real‐time PCR analysis was implemented with the SYBR Green qRT‐PCR Supermix kit (Takara, Japan) on the ABI 7500 System (Applied Biosystems). To ascertain the relative mRNA expression level of a gene, normalization was undertaken with GAPDH. The primers utilized for qRT‐PCR are listed in Table 1.

**TABLE 1:** The sequences of qRT‐PCR primers.

Primer name	Sequence (5′ to 3′)
RIOK1	TGCCTTGGAGTTCTTGAGAAAG
	TGATCTTGGCTAGACCGTTCTT
GAPDH	GAAAGCCTGCCGGTGACTAA
	GCATCACCCGGAGGAGAAA
PMS1	AGGAATGGCTAATTGTCTCCC
	TCTGGATAGACGCACTGCTT
SPDL1	ACTAAGCAGAAGCCATGGAC
	CATGCTTTCCTGCACACGTT
RAD18	ACAGCTCATTAAAAGGCACCA
	ACTAGCTTCAAGACGCATCC
BARD1	CACCACTTCACGATGCAGCCA
	AATCGACAGGCCGCAGACCA
SMARCA5	TGGCATCAATCTTGCGACT
	TCTGCCCAATTCTATGTGCT

### 2.5 Western Blotting

The tissue samples and cells were lysed using RIPA buffer (Beyotime, China) and the protein concentrations in the lysates were determined with a BCA kit (Beyotime, China). Equivalent amounts of protein were separated by 10% SDS‐PAGE and transferred to polyvinylidene fluoride membranes. After washing and blocking the membranes with sealing fluid, they were incubated with rabbit polyclonal anti‐RIOK1 (1: 2000 dilution, Abcam, ab88496), anti‐cyclin B1 (1:1000 dilution, Cell Signaling Technology, #4135), anti‐cyclin A2 (1:1000 dilution, Cell Signaling Technology, #67955), anti‐PMS1 (1: 1000 dilution, HUABIO, ER64635), anti‐SPDL1 (1: 1000 dilution, HUABIO, ER62926), anti‐RAD18 (1: 1000 dilution, HUABIO, ET1611‐32), anti‐BARD1 (1:1000 dilution, HUABIO, ER62796), anti‐SMARCA5 (1: 2000 dilution, Diagbio, db11595), anti‐β‐actin (1:1000 dilution, Cell Signaling Technology, #4970S), and anti‐GAPDH (1:1000 dilution, Cell Signaling Technology, #4135).

### 2.6 Real‐Time Cellular Analysis Assay

The RTCA assay was performed according to the manufacturer's instructions. Cells were harvested during the logarithmic growth phase and subjected to routine enzymatic dissociation. A cell suspension was prepared at a density of 1 × 10^5^ cells/mL, and 100 μL of this suspension was seeded into each well of the RTCA assay plates. Cell proliferation was monitored by recording the cell index at multiple time points after seeding.

### 2.7 Colony Formation Assay

Cells were seeded in 6‐well plates at low density (1000 cells/well) and cultured for 14 days. Cell colonies were fixed in 4% paraformaldehyde for 15 min, and 1 mL of 0.1% crystal violet was added (Sigma, USA) to each well for 30 min at room temperature for visualization.

### 2.8 Cell Cycle Assay

Cells were trypsinized, collected, and washed twice with cold PBS. Cells were fixed in 1 mL of DNA staining solution and 10 μL permeabilization solution for 30 min at room temperature. Flow cytometric quantification of the cell cycle was performed using a Dx‐FLEXS (Beckman) and analyzed using CytExpert for DxFLEX Software.

### 2.9 Mouse Tumor Xenograft Model

All animal experiments were conducted according to the guidelines for ethical review of laboratory animal welfare (China National Standard GB/T 35892–2018) and approved by the Animal Ethics Committee of NingboNo. 2 Hospital. A total of 1 × 10^7^ Huh7 cells (scrambled shRNA or RIOK1‐shRNA or RIOK1‐overexpression) in 200 μL PBS were injected subcutaneously into the right axilla of 4‐week‐old BALB/c nude mice (Vital River Laboratory Animal Technology Co. Ltd., Beijing, China). After a 28‐day observation period, the mice were euthanized under anesthesia. The tumors were excised and weighed.

### 2.10 RNA Sequencing Analysis

Huh7 cells were transfected with either a mock vector or a RIOK1‐overexpression construct (*n* = 3), and shCon or shRIOK1 (*n* = 3) were used for RNA sequencing analysis. The RNA‐Seq and subsequent analyses were performed using Shanghai Applied Protein Technology Co. Ltd (Shanghai, China).

### 2.11 Quantitative Proteomic Analysis by Tandem Mass Tag Technology

Tandem mass tag‐based quantitative proteomic analysis was conducted by Shanghai Applied Protein Technology Co Ltd. Initially, a total of 1 × 10^7^ cells were placed in SDT lysis buffer (4% w/v SDS, 100 mM Tris/HCl, pH 7.6, 0.1 M DTT) to extract the protein fraction. The protein concentration in the supernatant was determined using a BCA protein assay kit. Subsequently, an appropriate quantity of protein was obtained from each sample and subjected to trypsin digestion using filter‐aided proteome preparation (FASP). The resulting peptide levels were then quantified based on OD280. Subsequently, 100 μg of peptide from each sample was subjected to labeling using TMT Isobaric Mass Tagging Kits (Thermo Scientific, USA). The labeled peptides from each sample were then combined in equal proportions and fractionated using a High pH Reversed‐Phase Peptide Fractionation kit, following the manufacturer's instructions. LC–MS/MS analysis was conducted using a Q‐Exactive mass spectrometer (Thermo Scientific, USA) coupled with an Easy‐nLC 1000 instrument (Thermo Scientific, USA), as previously described [12]. Protein identification and quantitative analysis were carried out using Mascot 2.2 and Proteome Discoverer 1.4.

### 2.12 Statistical Analysis

All the experiments were performed in triplicate and repeated independently at least three times. The sample sizes and statistical methods are indicated in the corresponding figure legends. The data are represented as the mean ± SEM. All data plotting and statistical analyses were performed by GraphPad Prism 8 software. Differences were statistically significant at *p < 0.05, **p < 0.01, ***p < 0.001, and ****p < 0.0001.

The differential analysis of proteins is conducted using univariate statistical analysis (Student *t*‐test or Mann–Whitney *U*‐test, depending on the normality of data and homogeneity of variance). This project's threshold value for differential protein selection is *p* < 0.05 and |log2FC| ≥ 1.2. To perform differential analysis of transcriptomics data, microarray data were normalized and analyzed using the Limma R package; differentially expressed genes (DEGs) must meet the following criteria: |log2FC| > 2, and *p* < 0.05. The magnitude of the correlation between target genes and RIOK1 is assessed using the Pearson correlation coefficient.

By intersecting the differential genes and proteins from univariate statistics with similar patterns of change in expression, we utilized the MetScape website (https://www.metascape.org/gp/index.html) for function enrichment analysis [13]. MetScape integrates data resources from several authorities to construct enrichment networks, such as KEGG Pathway, GO Biological Processes, Reactome Gene Sets, Canonical Pathways, CORUM, WikiPathways, and PANTHER Pathway. All genes in the genome were used as the enrichment background. Terms with a *p*‐value < 0.01, minimum count of 3, and enrichment factor > 1.5 were collected and grouped into clusters based on their membership similarities. To further capture the relationships between the terms, a subset of enriched terms were selected and rendered as a network plot, where terms with a similarity > 0.3 are connected by edges. The network is visualized using Cytoscape, where each node represents a matched gene and is colored first by its cluster ID.

## 3 Results

### 3.1 RIOK1 Overexpressed In HCC Tissues and Associated With Poor Prognosis In HCC Patients

**FIGURE 1:**
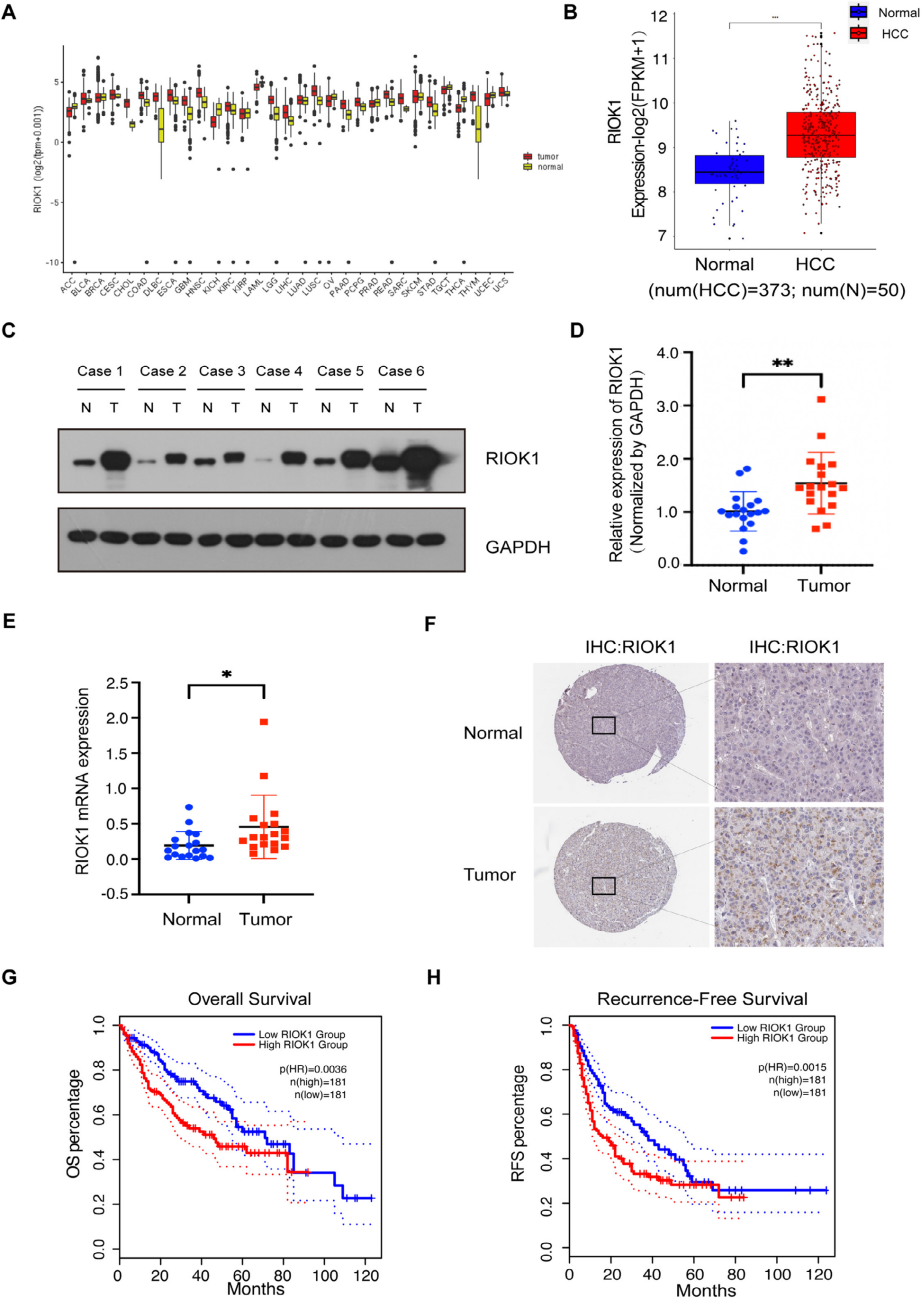
RIOK1 expression in HCC and Kaplan–Meier survival analysis of HCC patients. (A) The expression of RIOK1 in pan‐cancer was examined from the TCGA database. (B) The mRNA expression of RIOK1 in TCGA between liver cancer tissues and normal tissues. (C) The expression of RIOK1 in HCC and adjacent tissues was detected by Western blot. (D) Statistical analysis of the gray value in WB assay; **p < 0.01. (E) The expression of RIOK1 in HCC and adjacent tissues was examined by qRT‐PCR; *p < 0.05. (F) Representative H&E and immunohistochemical staining of RIOK1 in HCC and adjacent normal tissues. (G and H) Kaplan–Meier curves of OS and RFS in patients with HCC. HCC, hepatocellular carcinoma; qRT‐PCR, quantitative real‐time PCR; RIOK1, RIO kinase 1; TCGA, the Cancer Genome Atlas; WB, Western blot.

The expression of RIOK1 in pan‐cancer was validated by gene expression data of 31 cancer types from the Cancer Genome Atlas (TCGA) project and was generally elevated in cancers (Figure 1A). Among them, we compared the mRNA expression of RIOK1 between liver cancer and normal tissues using data from TCGA. The results indicated that the expression of RIOK1 in HCC tissues was higher that in normal tissues (Figure 1B). Furthermore, to confirm the expression level of RIOK1 in HCC tissues, we examined the mRNA and protein expression level of RIOK1 in HCC tissues and matched adjacent normal tissues by qRT‐PCR and Western blot. Results showed that compared with matched adjacent normal tissues (*N* = 18), RIOK1 has a significantly elevated expression in HCC tissues (*N* = 18). Consistently, immunohistochemistry results from the Human Protein Atlas (HPA) database indicated that RIOK1 was down‐expressed in normal tissues, but overexpressed in HCC tissues (Figure 1C–F). In addition, the Kaplan–Meier survival curves were used to describe the relationship between the expression of RIOK1 and overall survival (OS) /recurrence‐free survival (RFS) in HCC patients. These results exhibited that the high expression of RIOK1 had a significantly worse OS/RFS rate compared to the low RIOK1 group (log‐rank test, *p* < 0.05) (Figure 1G,H).

### 3.2 Knockdown of RIOK1 Inhibits HCC Cell Proliferation In Vivo and In Vitro

**FIGURE 2:**
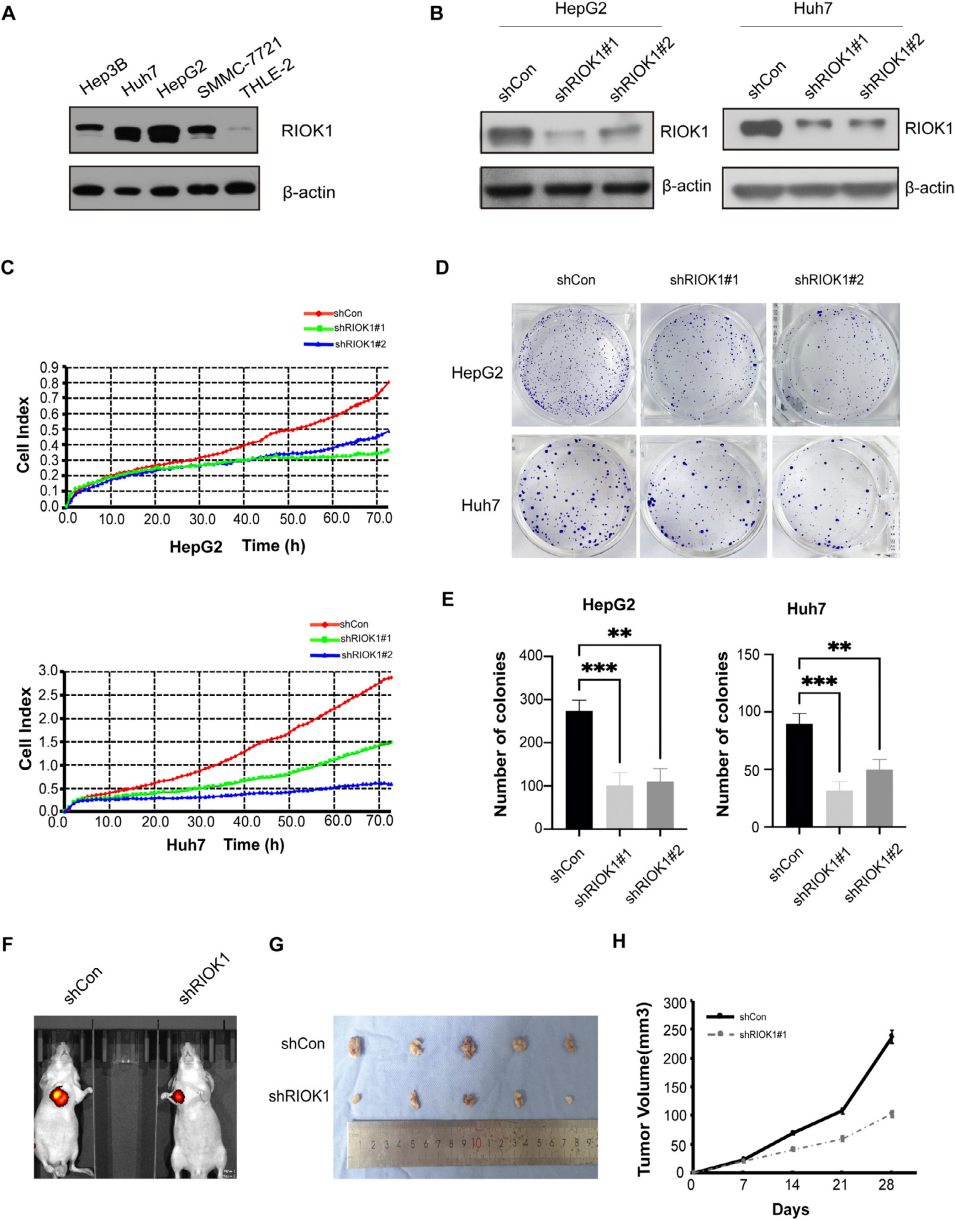
Knockdown of RIOK1 inhibits HCC cell proliferation in vitro and in vivo. (A) RIOK1 protein expression in HCC cell lines. (B) Western blot of RIOK1 in RIOK1 knockdown (shRIOK1#1 or shRIOK1#2) HepG2 or Huh7 cells. (C) Cell proliferation was detected by RTCA assays in HepG2 and Huh7 cell lines for up to 3 days. (D) Cell colony forming assay showed that knockdown of RIOK1 declined the cell growth of HepG2 and Huh7 cells. (E) Statistical analysis of the number in cell colony assay; All data are presented as the mean ± SEM from three independent experiments. **p < 0.01, ***p < 0.001. (F and G) Representative images of xenograft tumors excised from nude mice injected with shRNA‐RIOK1 or shCon HCC cells. (H) Comparison of tumor growth curves of the shRNA‐RIOK1 groups and the shCon group. HCC, hepatocellular carcinoma; qRT‐PCR, quantitative real‐time PCR; RIOK1, RIO kinase 1; TCGA, the Cancer Genome Atlas; WB, Western blot.

To elucidate the functions of RIOK1 in HCC, we first utilized a western blot assay to detect its expression across HCC cell lines. The results showed that RIOK1 was highly expressed in HCC cell lines, especially in HepG2 and Huh7, using THLE‐2 as a negative control (Figure 2A and Figure S1A). So we selected HepG2 and Huh7 cell lines for subsequent studies. Next, we constructed stable RIOK1 knockdown cell models by infecting HCC cell lines with lentiviruses containing two altered shRNA sequences against RIOK1 (shRIOK1#1 and shRIOK1#2) (Figure 2B and Figure S1B). A real‐time cellular analysis (RTCA) assay was used to test cell proliferation in HepG2 and Huh7 cells. The growth curves exhibited that knockdown of RIOK1 markedly inhibited the proliferation of HepG2 and Huh7 cells (Figure 2C). As shown in Figure 2D,E, the cell survival curves garnered from the clonogenic survival assay revealed that silencing of RIOK1 significantly weakened colony formation. Furthermore, to examine the role of RIOK1 in vivo, we subcutaneously engrafted stable shCon and shRIOK1 Huh7 cell lines into nude mice to establish xenograft models. By monitoring tumor volumes, we found that RIOK1 knockdown significantly inhibited tumor growth compared to the control group (Figure 2F–H). Taken together, these findings demonstrate that RIOK1 knockdown suppresses HCC proliferation both in vitro and in vivo.

### 3.3 RIOK1 Overexpression Promotes HCC Cell Proliferation In Vivo and In Vitro

**FIGURE 3:**
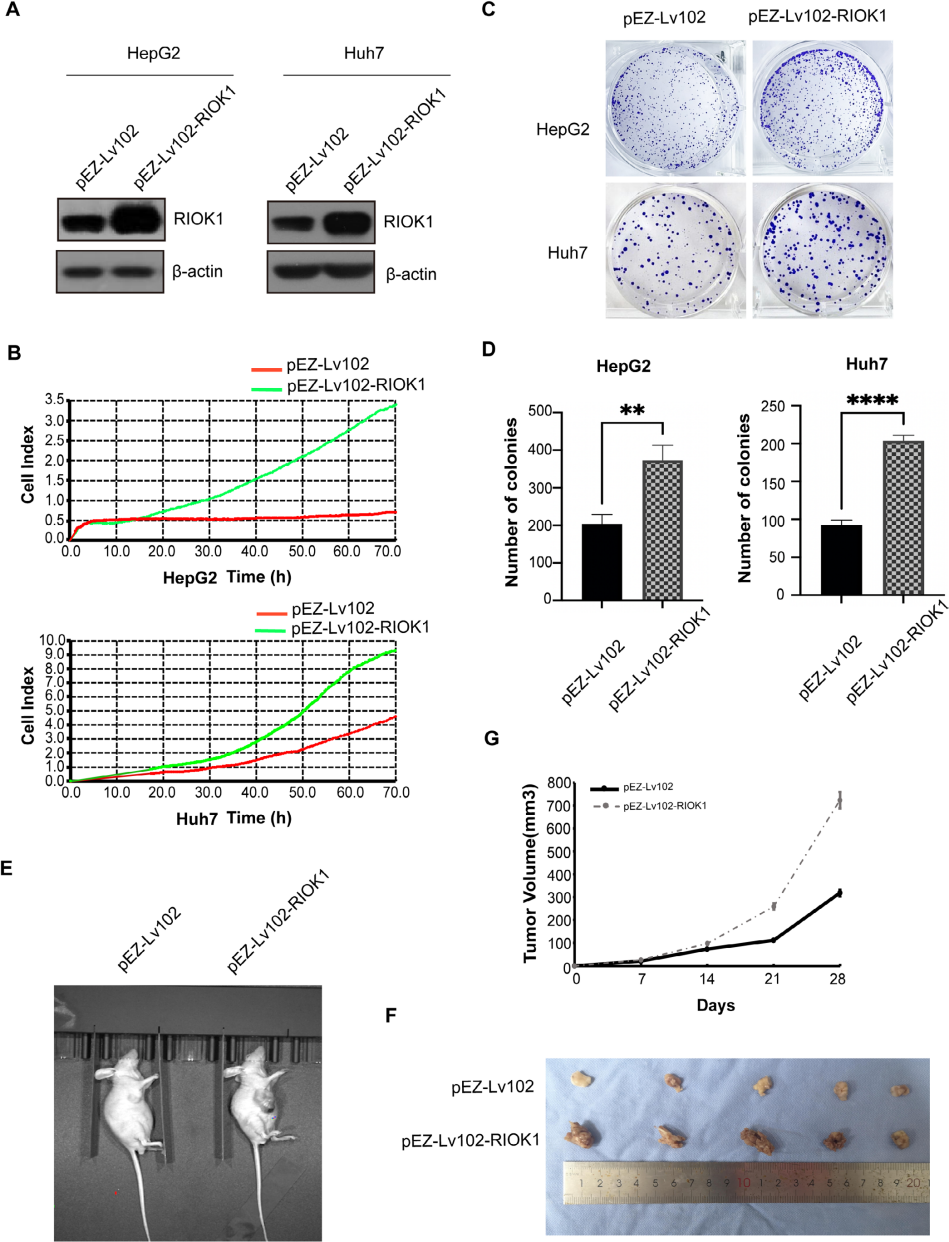
RIOK1 overexpression promotes HCC cell proliferation in vitro and in vivo. (A) Stable overexpression of RIOK1 in liver cancer cell lines was detected by Western blot. (B) Cell proliferation was detected by RTCA assays in HepG2 and Huh7 cell lines for up to 70 h. (C) Cell colony forming assay showed that overexpression of RIOK1 promoted the cell growth of HepG2 and Huh7 cells. (D) Statistical analysis of the number in cell colony assay; All data are presented as the mean ± SEM from three independent experiments. **p < 0.01, ****p < 0.0001. (E and F) Representative images of xenograft tumors excised from nude mice injected with RIOK1‐overexpressing (pEZ‐Lv102‐RIOK1) and vector (pEZ‐Lv102) Huh7 cells. (G) Comparison of tumor growth curves of the RIOK1‐overexpressing group and the control group. HCC, hepatocellular carcinoma; qRT‐PCR, quantitative real‐time PCR; RIOK1, RIO kinase 1; TCGA, the Cancer Genome Atlas; WB, Western blot.

To further determine the role of RIOK1 on HCC cell proliferation, we constructed HepG2 and Huh7 cell models stably overexpressing RIOK1 using lentiviral infection (Figure 3A and Figure S2A). RTCA assays revealed that enhanced growth profiles of HepG2 and Huh7 cells overexpressing RIOK1 compared to controls (Figure 3B). Colony formation assays provided additional evidence that overexpression of RIOK1 enhanced colony formation in HCC cells (Figure 3C,D), consistent with the antiproliferative effect of RIOK1 knockdown. We also examined the pro‐proliferative effects of RIOK1 in vivo by subcutaneous transplantation of RIOK1 overexpressing and vector Huh7 cells into nude mice. Overexpression of RIOK1 markedly accelerated tumor growth compared to controls (Figure 3E,F). Furthermore, the average tumor mass was significantly greater in the RIOK1 overexpression group compared to the vector group (Figure 3G). These results exhibited that high expression of RIOK1 promoted HCC cell proliferation in vivo and in vitro.

### 3.4 Knockdown of RIOK1 Inhibits HCC Cell Cycle

**FIGURE 4:**
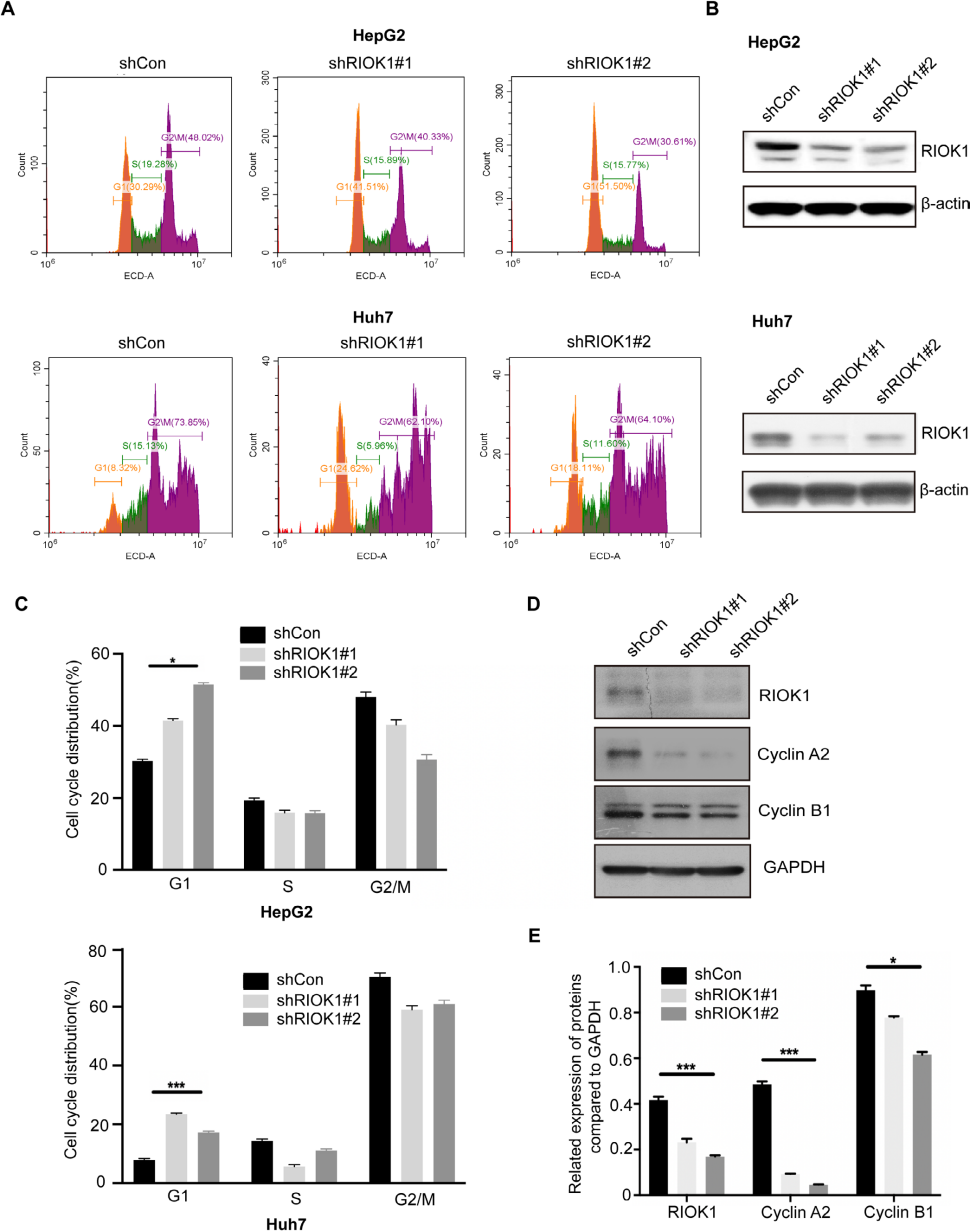
RIOK1 depletion affected the cell cycle in HCC cells. (A) Knockdown of RIOK1 resulted in G1‐phase cell cycle arrest in HepG2 and huh7 cells. (B) Stable knockdown of RIOK1 in HepG2 and Huh7 cell lines was detected by Western blot. (C) Statistical analysis of cell cycle. All data are presented as the mean ± SEM from three independent experiments. *p < 0.05, ***p < 0.001. (D) Western Blot analysis of cell cycle‐related proteins cyclin A2 and cyclin B1. (E) Statistical analysis of gray value in Western blot. *p < 0.05, ***p < 0.001. HCC, hepatocellular carcinoma; qRT‐PCR, quantitative real‐time PCR; RIOK1, RIO kinase 1; TCGA, the Cancer Genome Atlas; WB, Western blot.

To investigate the effects of RIOK1 on cell cycle progression, cell cycle assays were performed on HepG2 and Huh7 cells following RIOK1 silencing. The results showed an increased accumulation of cells in the G1 phase upon RIOK1 knockdown (Figure 4A–C and Figure S2B), indicating RIOK1 loss induces G1 cell cycle arrest. Western blot analysis further demonstrated that the key cell cycle regulatory proteins cyclin A2 and cyclin B1 were downregulated after RIOK1 knockdown (Figure 4D,E). Together, these data suggest that RIOK1 is required for cell cycle progression in HCC.

### 3.5 Integrated Analysis of Transcriptomic and Proteomic

To further elucidate the molecular mechanism of RIOK1 in HCC, we performed transcriptomic and proteomic analyses on Huh7 cells with RIOK1 knockdown or overexpression. A total of 10,588 genes were identified by RNA sequencing analysis. Using |fold change| ≥ 2 and FDR ≤ 0.05 as the detection criteria, we identified 1792, 2081, and 2246 DEGs in shRIOK1#1, shRIOK1#2, and RIOK1‐overexpression (RIOK1‐OE) cells, respectively. Among these genes, 952 downregulated and 840 upregulated DEGs were found in shRIOK1#1 cells, 1291 downregulated and 790 upregulated DEGs were found in shRIOK1#2 cells, and 1192 downregulated and 1054 upregulated DEGs were found in RIOK1‐OE cells. Proteomic analysis identified 6892, 7101, and 7043 proteins in shRIOK1#1, shRIOK1#2, and RIOK1‐OE cells, respectively. Differentially expressed proteins (DEPs) were identified by t‐test (*p* < 0.05). The results showed that 1662 downregulated and 1711 upregulated DEPs were found in the shRIOK1#1 cells, 2217 downregulated and 2235 upregulated DEPs were found in the shRIOK1#2 cells, and 1853 downregulated and 1949 upregulated DEPs were found in the RIOK1‐OE cells.

**FIGURE 5:**
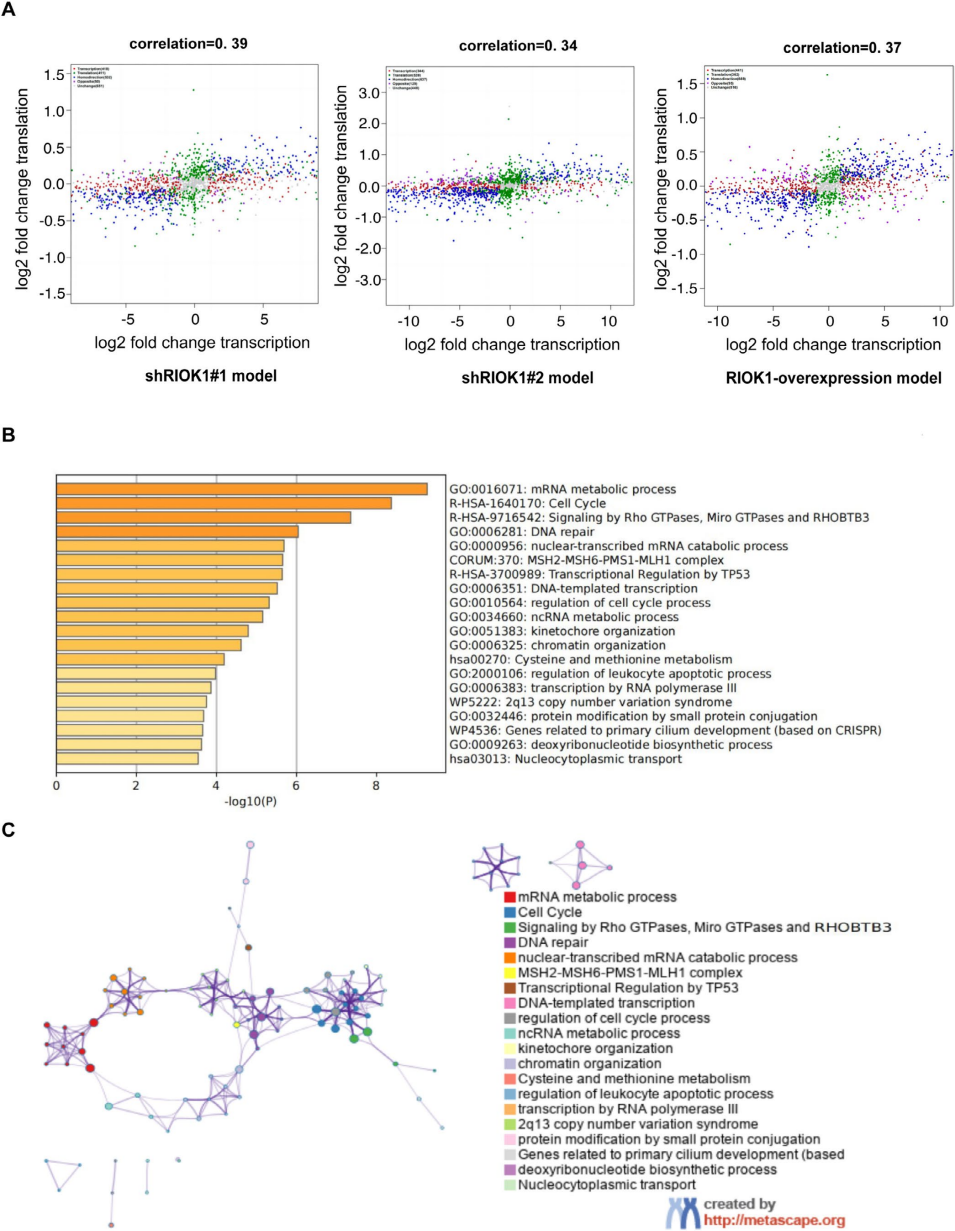
Proteo‐transcriptomics correlation. (A) Scatter plot of 9‐quadrant associate analyses of mRNA and proteins from log2FC. Gray dots (Unchange): No significant difference between transcriptomics and proteomics; Red dots (Transcription): Significant difference in transcriptomics and no significant difference in proteomics; Green dots (Translation): No significant difference in transcriptomics and significant difference in proteomics; Blue dots (Homodirection): Significant differences in both transcriptomics and proteomics with the same fold change trend; Purple dots (Opposite): Significant differences in both transcriptomics and proteomics, but with opposite trends in folding changes. (B) Heatmap of top 20 enriched terms across 250 positive genes identified from the concordant genes, colored by *p* values. (C) Networks of enriched terms are colored by cluster ID and nodes that share the same cluster are typically close. HCC, hepatocellular carcinoma; qRT‐PCR, quantitative real‐time PCR; RIOK1, RIO kinase 1; TCGA, the Cancer Genome Atlas; WB, Western blot.

An integrated analysis of the transcriptomic and proteomic data was performed to determine the correlation between gene and protein expression changes following RIOK1 knockdown or overexpression. First, correlation analysis was carried out on the mRNAs and proteins for each matched gene‐protein pair with Spearman's rank correlation coefficient. The overall correlations were 0.39 (shRIOK1#1), 0.34 (shRIOK1#2), and 0.37 (RIOK1‐OE) (Figure 5A), indicating there was a moderate concordance between the changes in mRNA and protein. However, some genes showed a divergence between mRNA and protein expression changes, which might be caused by posttranscriptional or translation‐level regulation. Then, “concordant genes” were screened from the multi‐omics data, referring to those genes that show consistent trends at both mRNA and protein levels. The number of concordant genes was 532, 637, and 669 in shRIOK1#1, shRIOK1#2, and RIOK1‐OE cells, respectively. Furthermore, 250 positive genes upregulated in cells with RIOK1 overexpression or downregulated in cells with RIOK1 deletion were identified from concordant genes. Based on these positive genes, we constructed an enrichment network associated with RIOK1 expression. The network consists of multiple clusters, each representing an enrichment pathway, and the nodes in the cluster are the matched genes (Figure 5B,C). Clusters with more shared genes are typically close to each other. Pathway and process enrichment analysis revealed that genes associated with RIOK1 expression were mainly involved in the mRNA metabolic process, cell cycle, signaling by Rho GTPases, Miro GTPases and RHOBTB3, DNA repair, and nuclear‐transcribed mRNA catabolic process. Among them, the cell cycle pathway and signaling by Rho GTPases, Miro GTPases and RHOBTB3 pathway are closely related and can jointly perform various cell biological functions such as cell proliferation and apoptosis. The complete list of significantly enriched pathways is provided in Table 2.

**TABLE 2:** Pathway and process enrichment analysis.

GO	Category	Description	Count	%	Log10 (*p*)	Log10 (*q*)
GO:0016071	GO biological processes	mRNA metabolic process	24	9.6	−9.3	−4.9
R‐HSA‐1640170	Reactome gene sets	Cell cycle	24	9.6	−8.4	−4.4
R‐HSA‐9716542	Reactome gene sets	Signaling by Rho GTPases, Miro GTPases and RHOBTB3	23	9.2	−7.4	−3.9
GO:0006281	GO biological processes	DNA repair	17	6.8	−6.0	−2.9
GO:0000956	GO biological processes	Nuclear‐transcribed mRNA catabolic	8	3.2	−5.7	−2.6
CORUM:370	CORUM	MSH2‐MSH6‐PMS1‐MLH1 complex	3	1.2	−5.7	−2.6
R‐HSA‐3700989	Reactome gene sets	Transcriptional Regulation by TP53	14	5.6	−5.6	−2.6
GO:0006351	GO biological processes	DNA‐templated transcription	18	7.2	−5.5	−2.5
GO:0010564	GO biological processes	Regulation of cell cycle process	20	8	−5.3	−2.3
GO:0034660	GO biological processes	ncRNA metabolic process	16	6.4	−5.2	−2.3
GO:0051383	GO biological processes	Kinetochore organization	4	1.6	−4.8	−2.0
GO:0006325	GO biological processes	Chromatin remodeling	17	6.8	−4.6	−1.8
hsa00270	KEGG Pathway	Cysteine and methionine metabolism	5	2	−4.2	−1.5
GO:2000106	GO Biological Processes	Regulation of leukocyte apoptotic process	6	2.4	−4.0	−1.3
GO:0006383	GO Biological Processes	Transcription by RNA polymerase III	4	1.6	−3.9	−1.2
WP5222	WikiPathways	2q13 copy number variation syndrome	5	2	−3.7	−1.1
GO:0032446	GO Biological Processes	Protein modification by small protein conjugation	17	6.8	−3.7	−1.1
WP4536	WikiPathways	Genes related to primary cilium development (based on CRISPR)	6	2.4	−3.7	−1.1
GO:0009263	GO Biological Processes	Deoxyribonucleotide biosynthetic process	3	1.2	−3.6	−1.1
hsa03013	KEGG Pathway	Nucleocytoplasmic transport	6	2.4	−3.5	−1.0

To better understand the function of RIOK1 in the HCC, we analyzed the pathway enrichment of DEGs between RIOK1‐high and RIOK1‐low patients in HCC and other cancers using the public database TCGA (Figure S3). The results showed that the genes potentially affected by RIOK1 in HCC are mainly enriched in the cell cycle, nucleocytoplasmic transport, ATP‐dependent chromatin remodeling, polycomb repressive complex, and DNA replication (Figure S3A), which was consistent with several pathways we obtained from the analysis of our multi‐omics data, such as the cell cycle, cytoplasmic nuclear translocation, and chromatin remodeling. RIOK1 may affect HCC occurrence and development through these pathways. Furthermore, compared with the results of pathway enrichment analysis for other cancers, we found that several pathways in HCC also share some commonalities with other cancers, including the cell cycle, DNA repair, the p53 pathway, and chromatin remodeling, which may be closely related to the promotion of cancer development by RIOK1 (Figure S3B–F).

### 3.6 Identification and Analysis of RIOK1‐Related Core Genes in the Cell Cycle and DNA Repair Pathways

**FIGURE 6:**
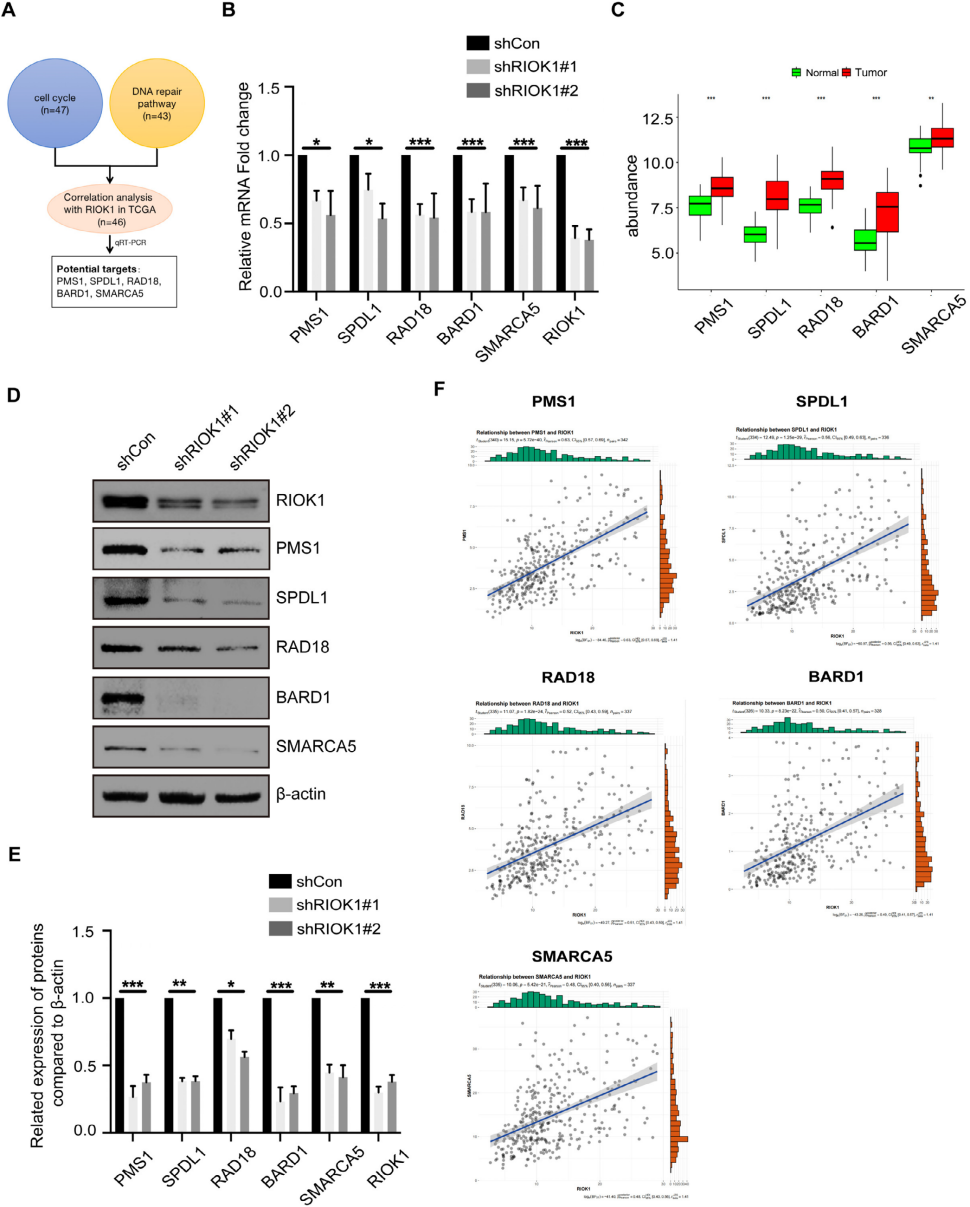
Analysis of RIOK1‐related core genes. (A) Schematic diagram of the RIOK1‐related core gene screen. (B) The mRNA expression level of five core genes with RIOK1 depletion. All data are presented as the mean ± SEM from three independent experiments. *p < 0.05, ***p < 0.001. (C) Differential expression of five core genes (PMS1, SPDL1, RAD18, BARD1, and SMARCA5) between tumor and normal tissues in the TCGA‐LIHC cohort. The asterisks above histograms indicate whether the two groups are statistically significantly different as assessed by one‐way ANOVA followed by a Tukey HSD test. **p < 0.01, ***p < 0.001. (D) Western blot analysis of five core genes after depletion of the RIOK1 gene. (E) Statistical analysis of gray value in Western blot. *p < 0.05, **p < 0.01, ***p < 0.001. (F) Correlation analysis between these five core genes and RIOK1 in TCGA‐LIHC cohort.; qRT‐PCR, quantitative real‐time PCR; RIOK1, RIO kinase 1; TCGA, the Cancer Genome Atlas; WB, Western blot.

**FIGURE 7:**
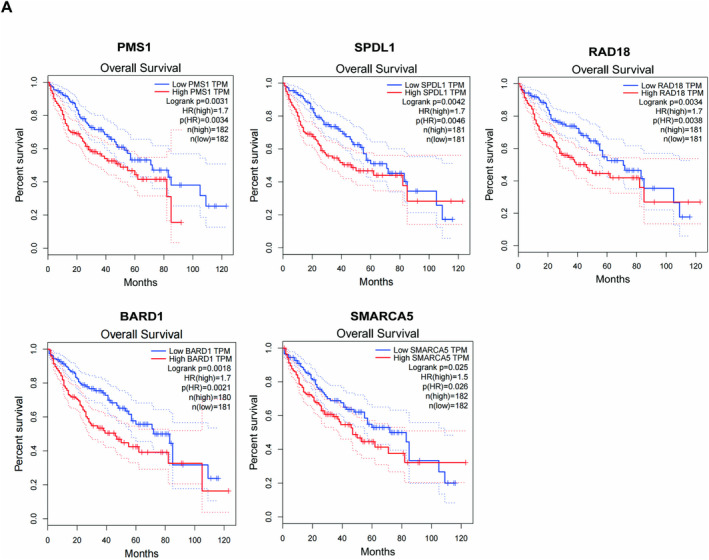
Predictive analysis of the RIOK1‐related five core genes signature: Kaplan–Meier curves for overall survival in the TCGA‐LIHC cohort. RIOK1, RIO kinase 1; TCGA, the Cancer Genome Atlas.

Our previous functional experiments demonstrated that RIOK1 plays a pivotal role in promoting the occurrence of hepatocellular carcinoma. Therefore, based on the results of the integrated analysis of transcriptomic and proteomic, we focused our investigation on the core genes regulated by RIOK1 in the cell cycle and DNA repair pathways and identified a total of 47 genes in the cell cycle and 43 genes in the DNA repair pathway. Then we combined them with the public data in the TCGA database and used a Pearson correlation coefficient greater than 0.5 as the screening criterion to identify genes that are more likely to be associated with RIOK1 expression, and ultimately obtained 46 target genes (Table 3). Moreover, we screened and validated these genes by qRT‐PCR and identified five potential effectors that may mediate the oncogenic effects of RIOK1 in HCC, namely PMS1, SPDL1, RAD18, BARD1, and SMARCA5 (Figure 6A). Silencing of RIOK1 significantly reduced the mRNA and protein expression levels of these five genes (Figure 6B,D,E). TCGA public data confirmed that RIOK1 and five core genes were upregulated in HCC tissues compared to normal tissues (Figure 6C). Furthermore, we observed positive correlations between RIOK1 and the five genes (Figure 6F). In addition, patients with high expression of these five genes had significantly shorter overall survival (Figure 7). In conclusion, based on the results of pathway and process enrichment analyses, we identified and validated five core genes regulated by RIOK1 in cell cycle and DNA repair pathways, which may cooperate with RIOK1 to regulate the occurrence of HCC.

**TABLE 3:** Genes highly associated with RIOK1 expression in cell cycle and DNA repair pathways.

Number	Gene	Correlation analysis with RIOK1 in TCGA (Pearson's correlation coefficient)
1	SMARCAL1	0.67
2	WDR33	0.65
3	ACTL6A	0.65
4	XRCC5	0.65
5	PNO1	0.64
6	UIMC1	0.64
7	PMS1	0.63
8	RAD1	0.63
9	WDR48	0.62
10	MSH2	0.62
11	SFPQ	0.61
12	ORC2	0.60
13	NUP155	0.58
14	RIF1	0.58
15	TSR1	0.58
16	TRIP12	0.57
17	SPDL1	0.56
18	PARP1	0.56
19	ACTR8	0.56
20	POLR2B	0.55
21	SETD2	0.55
22	XPO1	0.54
23	DYNC1LI1	0.54
24	EYA3	0.54
25	AHCTF1	0.53
26	MAD2L1	0.52
27	MCM6	0.52
28	RAD18	0.52
29	MLH1	0.51
30	CENPF	0.50
31	CKS1B	0.50
32	CLASP2	0.50
33	RBBP4	0.50
34	NDC1	0.50
35	TTF2	0.50
36	COPS4	0.50
37	BARD1	0.50
38	MSH6	0.50
39	WDR77	0.50
40	MCM2	0.49
41	RFC1	0.48
42	SPC25	0.48
43	SMARCA5	0.48
44	CLASP1	0.48
45	ANAPC4	0.48
46	FANCD2	0.48

## 4 Discussion

RIOK1 is a member of the atypical serine/threonine protein kinase family and possesses protein kinase and ATPase activities. It participates in the regulation of various cellular processes including cell proliferation, cell survival, and gene transcription [6, 14]. Previous studies had reported high expression of RIOK1 in various solid tumors, including lung cancer, breast cancer, and prostate cancer [7, 8, 9, 10]. And its high expression was correlated with worse overall survival and poor prognosis [6, 7, 10]. In this study, we found RIOK1 was also highly expressed in HCC and was associated with poor prognosis, which supports previous research findings. Further investigations have shown that abnormal activation of RIOK1 was linked to various biological functions in cancer. In NSCLC, the knockdown of RIOK1 substantially suppressed proliferation and metastasis [6, 7]. In HR‐negative breast cancer cells, overexpression of RIOK1 promoted cell growth and invasion by altering PI3K/AKT and MAPK/ERK signaling [10]. RIOK1 also cooperated with AKT1 to activate NF‐κB signaling, driving colorectal cancer progression [8]. Similarly, our current study demonstrated overexpression of RIOK1 promoted cell proliferation and viability in HCC cells, while depletion of RIOK1 suppressed cell proliferation and viability. Knockdown of RIOK1 could inhibit the HCC cell cycle progression and affect the expression of cyclin A2 and cyclin B1. These findings were in line with prior functional evidence in other cancers. In summary, our findings revealed a novel role for the kinase RIOK1 in promoting HCC development. Combined with earlier studies, these findings suggested that RIOK1 had the potential to be a pan‐cancer biomarker.

In this study, RIOK1 knockdown in cell lines was performed by lentiviral shRNA, a commonly used knockdown method that requires a relatively long knockdown time of more than 72 h. Given that RIOK1 is essential for ribosome production, knocking down RIOK1 over such a long period may well have reduced global protein production in a nonspecific manner (as a secondary effect), rather than a direct effect of RioK1 on gene transcription, pre‐mRNA splicing, and mRNA turnover, mRNA translation, and protein stability. Based on the above possibilities, more strategies for depleting RIOK1 for short periods could be adopted in the future, including N‐terminal degrons, which could completely deplete RIOK1 in a few hours rather than days, thus avoiding nonspecific consequences on intracellular protein synthesis/levels and allowing for more precise experimental results.

To further elucidate the molecular mechanisms underlying RIOK1's involvement in HCC pathogenesis, transcriptomic and proteomic analyses were conducted using RIOK1 cellular models. Pathway analysis implicated RIOK1 in regulating cell cycle and DNA damage response pathways in HCC, but further validation is needed, given that RIOK1 could exert its role in promoting cancer processes through other signaling pathways. For instance, RIOK1 has been reported to promote p53 degradation by phosphorylating G3BP2 in colorectal cancer [11]. Another report showed that RIOK1 promotes prostate cancer through the c‐myc/E2F pathway [9]. So, to further explore the molecular mechanisms of RIOK1 in HCC, those genes involved in the cell cycle and DNA damage response signaling pathways were screened and five potential downstream targets of RIOK1, exhibiting a significant positive correlation (Pearson's correlation coefficient greater than 0.45) with RIOK1 expression, were identified. These targets included PMS1, SPDL1, RAD18, BARD1, and SMARCA5. The roles of three genes (PMS1, SPDL1, and BARD1) in HCC have been reported among these genes. PMS1 is a key component of the DNA mismatch repair (MMR) pathway, which detects and repairs DNA mismatches to preserve genomic integrity [15, 16]. PMS1 mutations or loss of function are associated with various cancers, such as colorectal, gastric, and ovarian cancers [17, 18]. In HCC, PMS1 mutations or overexpression may impair the MMR pathway, leading to an increased accumulation of DNA mutations and genomic instability. Furthermore, PMS1 mutations can promote tumor development and progression by affecting cell cycle regulation, apoptosis, and other mechanisms. SPDL1 (also known as CCDC99), encodes a protein named hSpindly, which is a recently identified gene involved in cell cycle regulation [19]. SPDL1 is associated with various pathological activities in vivo, such as protein modification, cell mitosis, cell cycle, and tumorigenesis [20, 21, 22, 23, 24]. SPDL1 is an oncogene overexpressed in various malignancies and associated with poor prognosis [25, 26, 27, 28]. Abnormal expression of SPDL1 may cause genomic instability, which accelerates tumor progression, invasion, and metastasis [29, 30]. High expression of SPDL1 was correlated with poor OS and DFS in HCC patients [25]. BARD1 is a tumor‐related gene that interacts with BRCA1, forming a complex with E3 ubiquitin ligase [31]. This complex involves DNA damage repair, cell cycle regulation, and other processes that maintain genomic stability [31, 32, 33, 34]. A report showed that BARD1 gene hypomethylation (13.3%) was more prevalent in patients with liver cirrhosis‐induced HCC than in normal controls [35]. Except for these three genes, two genes, RAD18 and sMARCA5, linked to tumor progression and poor prognosis in several cancers were poorly studied in HCC. RAD18 is an E3 ubiquitin ligase that is involved in error‐prone replication, which can lead to carcinogenesis and tumor progression. Elevated RAD18 expression is linked to tumor progression and poor prognosis in several cancers, such as cervical and esophageal cancers [36, 37]. For instance, RAD18 enhances cell migration and invasion in cervical cancer by activating the JNK‐MMPs pathway [37]. SMARCA5 is an ATPase of the ISWI class of chromatin remodelers and has a vital role in tumor development and progression [38, 39, 40]. It is highly expressed in various tumors, such as leukemia [41, 42], glioma [43], breast [44, 45], and gastric cancers [46]. In a word, these five genes play important roles in cancer processing. However, the interaction between these five core genes and the potential target protein RIOK1 is still unclear. Further study on the functional relationship of these genes with RIOK1 would help reveal the specific mechanism of RIOK1 in HCC development and progression.

This study demonstrated the potential of RIOK1 as a novel therapeutic target for HCC, but it also faces several challenges that need to be addressed in future research. One of the challenges is whether the five core genes closely related to RIOK1 identified in this paper are expected to be new targets for RIOK1. Another challenge is elucidating the specific molecular mechanisms of the downstream signaling pathways and regulatory networks mediated by RIOK1 in HCC. In conclusion, this study reveals that RIOK1 may be a novel target for treating HCC, but its clinical application requires further study.

## Author Contributions

**Chunyan Ruan:** conceptualization (lead), data curation (equal), funding acquisition (equal), project administration (lead), writing – original draft (lead). **Tianyu Shang:** data curation (equal), funding acquisition (equal), methodology (equal). **Sijia Zhang:** data curation (equal), methodology (equal), software (equal). **Wenhong Ru:** investigation (equal), methodology (equal), writing – review and editing (equal). **Yuefeng Yang:** data curation (equal), funding acquisition (supporting). **Yi Shen:** project administration (equal), writing – review and editing (equal).

## Ethics Statement

Approval of the research protocol by an Institutional Review Board: Human Research Ethics Committee, Ningbo No. 2 Hospital.

Animal studies: The animal experiments were approved by the Ningbo Institute of Life and Health Industry IACUC Committee.

## Consent

HCC tissue samples were obtained from Ningbo Human Biobank, and informed consent was obtained from all participants.

## Conflicts of Interest

The authors declare no conflicts of interest.

## Supporting information


Data S1.

## Data Availability

The data that support the findings of this study are available from the corresponding author upon reasonable request.
