# Supplementary material for: RIOK1: A Novel Oncogenic Driver in Hepatocellular Carcinoma
Source: Cancer Med. 2025 Jan 25;14(3):e70597. doi: 10.1002/cam4.70597 (PMC11761428; doi:10.1002/cam4.70597)

## Supplementary Materials

**Figure S1. Statistical analysis of gray value in Western blot.** (A) The RIOK1 protein expression in HCC cell lines. (B) The efficiency of knockdown of RIOK1 (shRIOK1#1 or shRIOK1#2) in HepG2 or Huh7 cells.


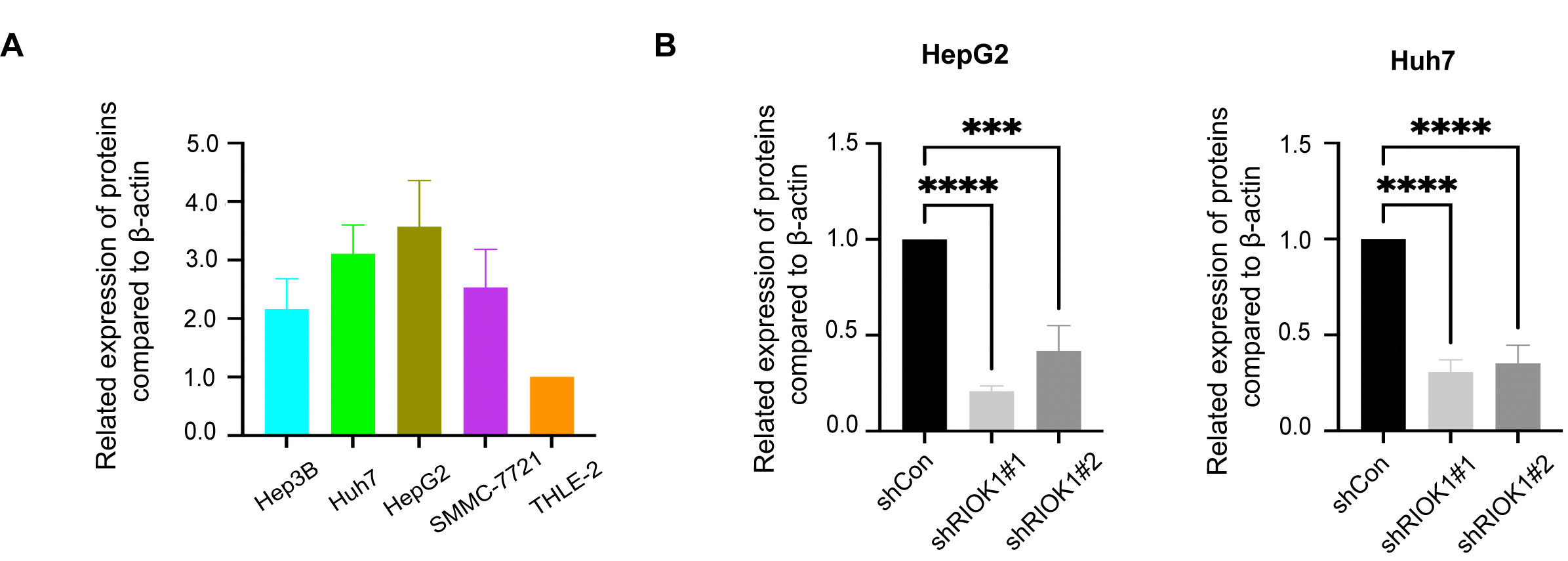


**Figure S2. Statistical analysis of gray value in Western blot.** (A) The overexpression levels of RIOK1 in HepG2 and Huh7 cells. (B) The efficiency of knockdown of RIOK1 (shRIOK1#1 or shRIOK1#2) in HepG2 or Huh7 cells.


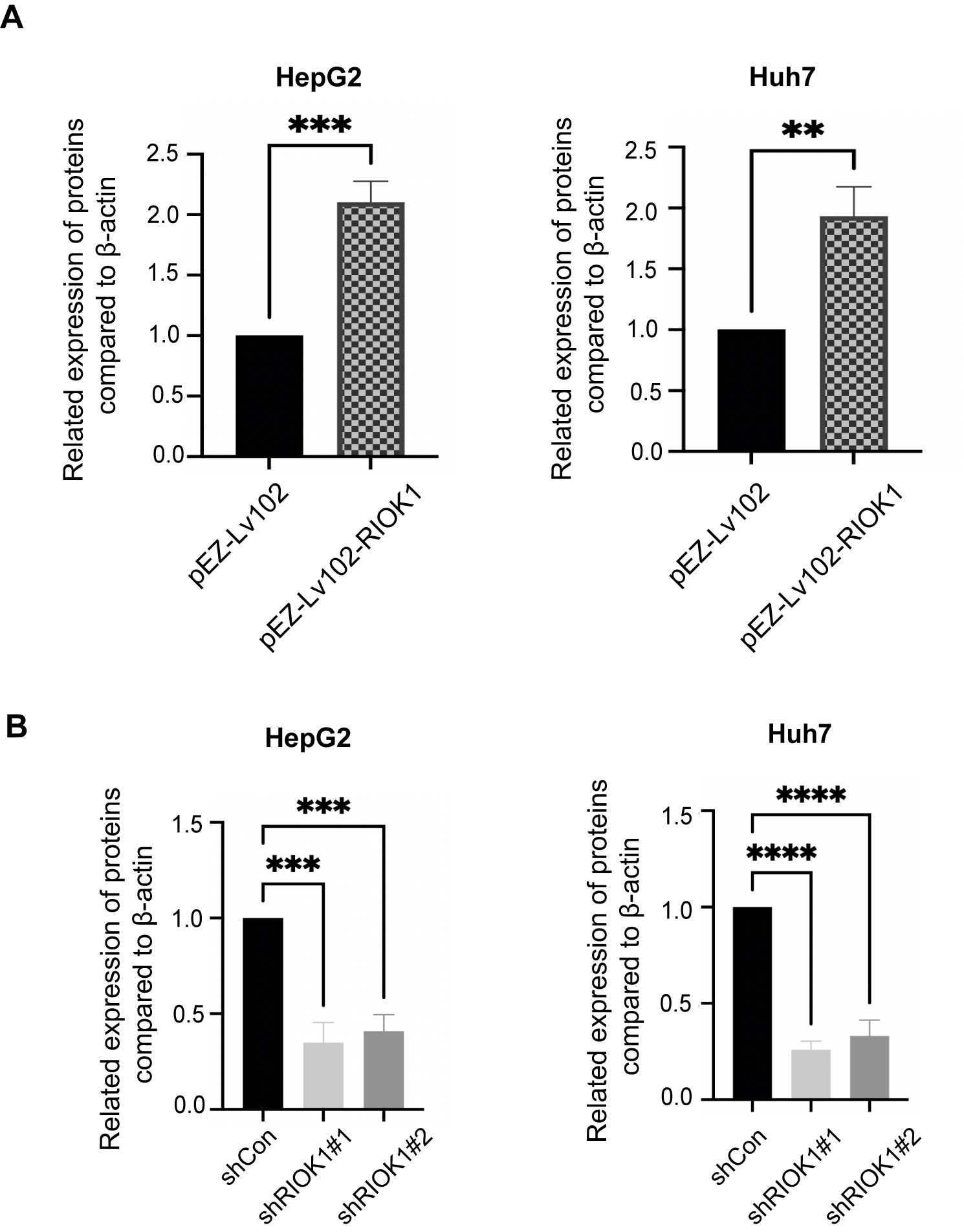


**Figure S3.** **KEGG analysis of DEGs between RIOK1-high and RIOK1-low patients in TCGA database.**


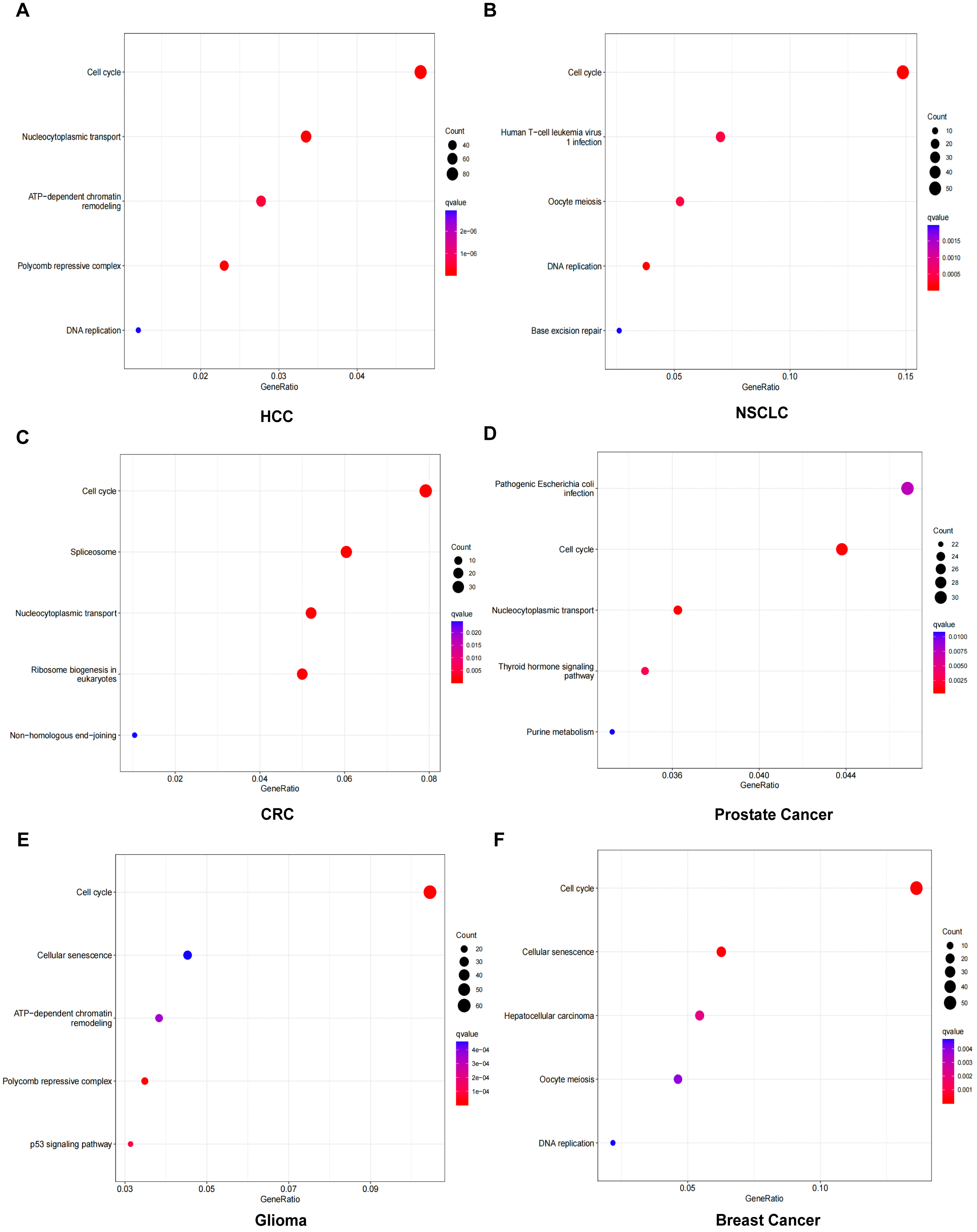

Supplement: Supplementary file 1 — Data S1. [file CAM4-14-e70597-s001.docx]
